# Multiresponse Optimization of Pomegranate Peel Extraction by Statistical versus Artificial Intelligence: Predictive Approach for Foodborne Bacterial Pathogen Inactivation

**DOI:** 10.1155/2019/1542615

**Published:** 2019-10-13

**Authors:** Mariam Fourati, Slim Smaoui, Karim Ennouri, Hajer Ben Hlima, Khaoula Elhadef, Ahlem Chakchouk-Mtibaa, Imen Sellem, Lotfi Mellouli

**Affiliations:** ^1^Laboratory of Microorganisms and Biomolecules, Center of Biotechnology of Sfax, University of Sfax, Road of Sidi Mansour Km 6, P. O. Box 1177, Sfax 3018, Tunisia; ^2^Algae Biotechnology Unit, Biological Engineering Department, National School of Engineers of Sfax, University of Sfax, Sfax 3038, Tunisia

## Abstract

Pomegranate (*Punica granatum* L.) peel is a potential source of polyphenols known for their activity against foodborne pathogen bacteria. In this study, the effects of pomegranate peel extraction time (10–60 min), agitation speed (120–180 rpm), and solvent/solid ratio (10–30) on phytochemical content and antibacterial activity were determined. Response surface methodology (RSM) and artificial neural network (ANN) methods were used, respectively, for multiresponse optimization and predictive modelling. Compared with the original conditions, the total phenolic content (TPC), the total flavonoid content (TFC), and the total anthocyanin content (TAC) increased by 56.22, 63.47, and 64.6%, respectively. Defined by minimal inhibitory concentration (MIC), the maximum of antibacterial activity was higher than that from preoptimized conditions. With an extraction time of 11 min, an agitation speed 125 rpm, and a solvent/solid ratio of 12, anti-*S*. *aureus* activity remarkably decreased from 1.56 to 0.171 mg/mL. Model comparisons through the coefficient of determination (*R*^2^) and mean square error (MSE) showed that ANN models were better than the RSM model in predicting the photochemical content and antibacterial activity. To explore the mode of action of the pomegranate peel extract (PPE) at optimal conditions against *S. aureus* and *S. enterica*, Chapman and Xylose Lysine Deoxycholate broth media were artificially contaminated at 10^4^ CFU/mL. By using statistical approach, linear (ANOVA), and general (ANCOVA) models, PPE was demonstrated to control the two dominant foodborne pathogens by suppressing bacterial growth.

## 1. Introduction

Biological wastes, annually generated in important amounts from the agro-food industries, arouse a significant disposal burden when they are directly disposed to soil or landfill under poor management [1, 2]. In this line, the fruit processing industry, such as pomegranate, implies the raw material transformation to increase the added-value products [3–5]. Therefore, a huge amount of solid waste was generated. For example, for the pomegranate processing, approximately 500 g/kg of the pomegranate fruit composed from inedible portion, i.e., the peel and the remaining half has about 400 g/kg of juice and 100 g/kg of seeds [6]. Obviously, they still have notable content of polyphenols such as flavonoids, anthocyanins, hydroxybenzoic acids, hydroxycinnamic acids, and tannins [7–10] with beneficial effects on human health. Reported data demonstrate that phenolic compounds from pomegranate peel have diverse beneficial properties to health including anticancer activity [11–14], antidiarrheal activity [15], higher free radical scavenging activities [16], and strong antimicrobial activity [13, 17, 18]. In this context, several studies have reported the efficacy of pomegranate peel extracts (PPEs) to inhibit the growth of Gram-positive and Gram-negative bacteria, which are foodborne pathogens, spoilage bacteria, and human pathogens [19–23].

The extraction of pomegranate peels was primarily conducted for separating the phenolic compounds from the plant tissues. In this regard, the polarity of the solvent, extraction temperature, solid–solvent ratio, and particle size are influential parameters for extraction process [24–26]. Extraction of phenolic compounds from pomegranate peel has been reported [27–31], and in the most of these works, organic solvents such as methanol and acetone came up as suitable extraction solvents to reach good yields. However, environmentally benign and nontoxic food-grade organic solvents, such as ethanol, are recommended by the US Food and Drug Administration for extraction purposes [26, 32, 33].

As a result of the inability of conventional single variable optimization in the assessment of the individual and combined interactions between the process parameters, using statistical modelling techniques is better and increasingly common way. One such approach is the response surface methodology (RSM), a collection of statistical and mathematical techniques useful in many engineering applications, was demonstrated to be able to optimize a process with a minimal amount of experimental data. As a statistical tool, RSM can model the impact of various process factors, both individually and through their cumulative interactions, providing an indication of best combination of parameters and response prediction [34]. In recent years, artificial neural network (ANN) is finding increasing use as a predictive tool in an extensive range of disciplines because of its ability to employ learning algorithms. ANN discerns also input–output relationships for complex and nonlinear multifactor systems using the generic structure and ability to learn from historical data [35, 36]. Recently, RSM and ANN methods have been used jointly for both modelling and optimizing natural product extraction processes and biological activities, and the obtained models showed strong correlation with experimental results [35, 36].

On the other hand, despite the intensive research activities carried out in the past decades, it was estimated that less than 10% of the known plant species in the world has been studied for antimicrobial activities, and data are lacking regarding their compositions and detailed antimicrobial mechanisms [37]. In this line, studies on the understanding of the mechanism of natural antimicrobial action, such as PPE, by the development of predictive mathematical models are still scarce. Furthermore, predictive inactivation models have been developed in liquid laboratory media that can mimic the microbial environment [38, 39].

As part of further research work pertaining to the exploration of the bioactive compounds and biological control against foodborne bacterial pathogens, in this study, PPE was optimized using RSM and ANN techniques to enhance simultaneously the total phenolic content (TPC), total flavonoid content (TFC), total anthocyanin content (TAC), and anti-*Staphylococcus aureus* and anti-*Salmonella enterica* subsp. *enterica* serovar *Typhimurium* activities. Equally, by skillfully using statistical approach (linear (ANOVA) and general (ANCOVA) models), results from this study provide insight into PPE effectiveness against *S. aureus* and *S. enterica*.

To the best of our knowledge, data on (i) the comparison of RSM and ANN techniques for pomegranate peel optimization for bioactive compounds and biological control and (ii) intensive investigation of the inactivation mode against two dominant foodborne pathogens are not available in the literature.

## 2. Materials and Methods

### 2.1. Plant Material

Pomegranate fruits (*Punica granatum* L.) were obtained during commercial harvest in November 2017 from local farms of Sfax (N: 34.4426°, E: 10.4537°), a central coastal region in Tunisia. Peels were cleaned, washed with running water, air-dried under ambient conditions, and reduced to a fine powder using an electric grinder (Moulinex, France) to get 40-mesh size powder.

### 2.2. Extraction Conditions

Pomegranate peel powders were subjected to extraction through the maceration technique with ethanol (Novachim, Bucharest, Romania) as the solvent. Using a shaker (Heidolph, Unimax 2010; Elektro GmbH, Kelheim, Germany), extractions were performed in Erlenmeyer flasks (Schott Duran, Voor't labo, Eeklo, Belgium) containing 2.0 g of the pomegranate peel powder sample. All extractions were performed at 20°C (cold extraction) for different extraction time (*X*1: 10–60 min), agitation speed (*X*2: 120–180 rpm) and solvent/solid ratio (*X*3: 10–30). Extraction time and agitation speed were controlled from the panel of the instruments. After extraction, each mixture was centrifuged (Sorvall Biofuge Stratos, ThermoScientific, Hanau, Germany) at 13,000×*g* for 10 min at 4°C. For removal of peel particles, the supernatant was filtered through cotton wool and Whatman paper No. 1 (Whatman Ltd, England) and then collected. Each extraction mixture was evaporated in a rotary evaporator (Laborota 4000, Heidolph, Milan, Italy) at 40°C obtaining a yellow–brown residue from peels that were immediately analyzed. PPEs were weighed to calculate the recovery extraction (%). The extraction procedure was carried out in triplicates.

### 2.3. Phytochemical Study of PPE

#### 2.3.1. Quantification of the TPC

TPCs from PPE were quantified by using the Folin–Ciocalteu method [40] adapted for a 96-well plate assay. PPEs were evaluated at the final concentration of 100 *μ*g/mL. 100 *μ*L of 0.2 N Folin–Ciocalteu reagent (Sigma-Aldrich GmbH, Steinheim, Germany) was added to a 96-well plate (SPL Life Sciences, Pocheon, Gyeonggi, South Korea) containing 20 *μ*L of each PPE with 80% ethanol and kept for 5 min in darkness at room temperature. 80 *μ*L of sodium carbonate at 75 g/L (Scharlau, Barcelona, Spain) was added to each well, and the plate was then incubated for 30 min at room temperature with slightly shaking in the darkness. Absorbance at 725 nm was measured in the spectrophotometer (PG Instrument Ltd. Model T60, United Kingdom). Gallic acid at 0–200 *μ*g/L (Sigma-Aldrich GmbH, Steinheim, Germany) was applied as standard. Results were expressed as mgGA equivalents/g dry sample (mg GAE/g) using the linear equation based on the calibration curve. Each assay was carried out in triplicates.

#### 2.3.2. Quantification of the TFC

TFCs in PPE samples were determined using the AlCl_3_ colorimetric method [41] adapted to 96-well plate. In brief, 100 *μ*L of 2% AlCl_3_ (Scharlau, Barcelona, Spain) was added to 100 *μ*L of each PPE in ethanol, and the plate was then incubated for 15 min at room temperature in the dark. The absorbance was determined at 430 nm. PPEs were evaluated at the final concentration of 100 *μ*g/mL. Quercetin at 0–50 *μ*g/mL (Sigma-Aldrich GmbH, Steinheim, Germany) was used as the standard. The total flavonoid contents were expressed as milligrams of quercetin equivalents mg QE/g of PPE. All the experiments were carried out in triplicates.

#### 2.3.3. Quantification of the TAC

TAC was evaluated by the pH differential method using two buffer systems: 25 mM potassium chloride (Sigma-Aldrich GmbH, Steinheim, Germany) solution (pH 1.0) and 0.4 M sodium acetate (Loba Chemie Pvt. Ltd., Mumbai, India) buffer (pH 4.5). PPEs were mixed with the corresponding buffers, and absorbances were determined simultaneously at 510 and 700 nm, respectively, after 15 min of incubation at 23°C [42].

(1) $$\begin{array}{c} A=\left(A510\;-\;A700\right)\text{ pH }1.0-\left(A510\;-\;A700\right)\text{ pH }4.5. \end{array}$$

TAC, expressed as mg of cyanidin-3-glucoside equivalents per 100 g of PPE (mg cy-3-glu/100 g), had been calculated as follows:

(2) $$\begin{array}{c} \text{TAC}=\frac{\left(A\times \text{MW}\times 1000\right)}{\varepsilon }, \end{array}$$

where *A*: absorbance; MW: molecular weight (449.2 g/mol); and *ε*: molar absorptivity of cyanidin-3-glucoside (26,900 M^−1^·cm^−1^).

### 2.4. Antibacterial Assays

#### 2.4.1. Bacterial Strains and Culture Conditions

Target bacteria strains were obtained from international culture collections (ATCC). They included Gram-positive bacterium: *Staphylococcus aureus* ATCC 6538 and a Gram-negative bacterium: *Salmonella enterica* subsp. *enterica serovar Typhimurium* ATCC 14028. *S. aureus* ATCC 6538 was grown on Chapman medium (Oxoid, Hampshire, United Kingdom), and *S. enterica* ATCC 14028 was cultivated on Xylose Lysine Deoxycholate (XLD, Oxoid CM0469, Basingstoke, United Kingdom) at 37°C for 24 h. For antagonist tests, the final inoculum concentration used for each indicator bacterium was 10^6^ CFU/ml [43].

#### 2.4.2. Determination of Minimum Inhibitory Concentrations (MICs)

The MIC of each PPE was determined against *S. aureus* ATCC 6538 and S*. enterica* ATCC 14028. The test was performed in sterile 96-well microplates with a final volume in each microplate well of 100 *μ*l. A stock solution of each extract, ranged between 0.0487 and 6.24 mg/mL, was prepared. To each test well, 10 *μ*l of cell suspension was added to final inoculum concentration of 10^6^ CFU/ml of bacterium. Positive growth control well consisted of *S. aureus* ATCC 6538 and *S. enterica* ATCC 14028, respectively, growth in Chapman and XLD media. Dimethyl sulfoxide (DMSO) (Loba Chemie Pvt. Ltd., Mumbai, India)/water (1/9) was used as the negative control. The plates were then covered with sterile plate covers and incubated for 24 h [44]. MIC was defined as the lowest concentration of each extract in which the microorganism did not demonstrate visible growth after incubation. As an indicator of microorganism growth, 25 *μ*l of Thiazolyl Blue Tetrazolium Bromide (MTT) (Sigma-Aldrich, Taufkirchen, Germany), indicator solution (0.5 mg/mL) dissolved in sterile water was added to the wells and incubated at 30 min. The colourless tetrazolium salt acts as an electron acceptor and was reduced to a red-coloured formazan product by biologically active organisms. Where microbial growth was inhibited, the solution in the well remained clear after incubation with MTT. The determinations of MIC values were done in triplicates.

### 2.5. Box–Behnken Design and Optimization by RSM

The extraction methodology was developed following the Box–Behnken design, and RSM was used to analyze the relationship between the measured responses and the individual and combined effects. Significant variables were optimized for enhanced phytochemical contents (TPC, TFC, and TAC), and anti-*S. aureus* and anti-*S. enterica* activities employing the Box–Behnken design [45].

The extraction time, agitation speed, and solvent/solid ratio were analyzed at three levels low, medium, and high coded as (−1), (0), and (+1), respectively, in fifteen runs. These variables: extraction time (*X*1: −1 : 10; 0 : 35; +1 : 60 min), agitation speed (*X*2: −1 : 120; 0 : 150; +1 : 180 rpm), and solvent/solid ratio (*X*3: −1 : 10; 0 : 20; +1 : 30) were selected for studying the effect and significance on TPC, TFC, TAC and the antibacterial activity.

The dummy variables were used to calculate the standard error. Each run was carried out in three replicates. The behavior of the system was explained by a second-order polynomial equation:

(3) $$\begin{array}{c} Y=\beta_{0}+\sum \beta_{i}X_{i}+\sum \beta_{ij}X_{i}X_{j}+\sum \beta_{ii}X_{i}^{2}, \end{array}$$

where *Y* is the predicted response, *β*_0_ is the offset term, *β*_*i*_ is the linear effect, βii is the squared effect, *β*_*ij*_ is the interaction effect, and *X*_*i*_ is the coded value of independent variables under study. This design was used to evaluate the main effects, interaction effects, and quadratic effects. It is also used to optimize the levels of parameters for enhancing the five responses. The statistical software Minitab 15.0 version was used for the experimental design and data analysis. Three-dimensional response surface plots were drawn to illustrate the relationship between the responses and the experimental levels of each independent variable. Optima levels of the variables for the maximum TPC, TFC, TAC, and the antibacterial activity was determined by the response optimizer tool of the software.

### 2.6. ANN-Based Modelling

To forecast nonlinear processes via neural networks, the network type Multilayered Perceptron (MLP) was used [46, 47]. These analyses can be used to generate a system that would support forecast procedures. Modelling of the MLP network was achieved via tanh.

In this study, in order to guarantee the simplest network design, the networks were constructed with one hidden layer: the input layer consists of three neurons (*X*1, *X*2, and *X*3), while the output layer had five neurons (TPC, TFC, TAC, anti-*S. aureus*, and anti-*S. enterica* activities). To measure the performance of ANN models for approximating the desired output of the five responses, the coefficient of determination (*R*^2^) was calculated. In fact, *R*^2^ can be defined as a standard criterion for the evaluation of statistical performance and employed to verify the accuracy of the predictive ability of the assembled models [48]. The training data set was applied to train the ANN to locate the overall comprehensive model between its inputs and outputs. The test data were used to validate and corroborate the predictive value of the expanded networks.

Neural Network module of STATISTICA 8.0 software was used in modelling the ANN. The data were categorized into two parts: training (80%) and testing (20%). In the network, there were three inputs and one output, corresponding to the TPC, TFC, TAC, and the antibacterial activity. The hidden neurons were optimized by building various MLP with hidden nodes from 1 to 10. Networks with hidden nodes greater than 10 were not developed because of the predictive capabilities decreasing with the decrease in the number of intermediate units.

### 2.7. Time-Kill Assay of PPE on Viable Counts of *S. aureus* ATCC 6538 and *S. enterica* ATCC 14028

The PPE effect on the inhibition of *S. aureus* ATCC 6538 and *S. enterica* ATCC 14028 was assessed by sequential sampling and counting viable bacteria, respectively, in the Chapman and the XLD broth. Firstly, *S. aureus* ATCC 6538 and *S. enterica* ATCC 14028 growth reached the beginning of the exponential phase (∼10^4^ CFU/ml). After three hours of incubation time, PPEs were added separately in four different concentrations (0.171, 0.342, 0.684, and 1.368 mg/mL for *S. aureus* and 0.555, 1.11, 2.22, and 4.44 for *S. enterica*) then incubated 37°C for 24 hours. At various points of incubation time: 0, 1, 2, 3, 4, 5, 6, 7, 8, 9, 10, 11, 12, 14, 16, 18, 20, 22, 24, and 26 hours, the numbers of CFU were determined by plating the samples on Chapman agar for *S. aureus* ATCC 6538 and XLD agar for *S. enterica* ATCC 14028 and then counting of the colonies that appeared. Controls were prepared under the same experimental conditions as mentioned above but without PPE addition. Each test was performed in triplicates.

### 2.8. Statistical Analysis

Measurements were carried out in triplicates and repeated three times. A one-way analysis of variance (ANOVA) and Tukey's post-hoc test were performed to determine significant differences between the responses using the Statistical Package for the Social Sciences (SPSS) software (SPSS Ltd. Woking, United Kingdom). Means and standard errors were calculated. Differences among the mean values of the various responses were determined by the least significant difference test. A probability level of *p* < 0.05 was used in testing the statistical significance of all the experimental data.

Plate count data were converted to logarithms prior to their statistical treatment. Linear mixed models assuming the error to compare CFU values among treatments with different time periods were used. Mixed models were fitted using SPSS 19 and followed by post hoc contrasts through the origin. The interpretation of the statistical output of a mixed model requires an understanding of how to explain the relationships among the fixed and random effects.

## 3. Results and Discussion

The effect of extraction time, agitation speed, and solvent/solid ratio on the recovery extraction of PPE was shown in Table 1. Recovery extraction of different experimental runs ranged between 14.4 ± 0.51 and 19.98 ± 0.95%. Among the fifteen runs, the highest (*p* < 0.05) PPE recovery extraction was recorded in run 2 with a value of 19.98 ± 0.95%. A similar trend has been observed by Malviya et al. [49] studying the effect of using different solvents on the extraction yield from pomegranate peels. In fact, these authors reported that recovery extraction was about 15%.

To develop an empirical model, an experimental design has been formulated by examining the interaction of different associated parameters *X*1 (time of extraction), *X*2 (agitation speed), and *X*3 (solvent/solid ratio). The design arrangement and experimental results of the extraction are shown in Table 1. In order to evaluate the fitness of response function, a total of 15 designed experiments were conducted for optimizing *X*1, *X*2, and *X*3 in a multivariable system. According to equation (3), *Y*_TPC_, *Y*_TFC_, *Y*_TAC_, *Y*_anti-*S. aureus*_, and *Y*_anti-*S. enterica*_ were calculated into the generalized model. Equally, the linear and quadratic effects of independent variables (*X*1, *X*2, and *X*3) of the multiple regression coefficients were calculated, and their interactions were also analyzed for regression coefficients in the RSM study.

### 3.1. Model Fitting and Analysis of Response Surfaces

The adequacy and fitness of the models were judged by the lack-of-fit significance and *R*^2^. For all responses, the statistical analysis indicated that the proposed model was adequate, possessing no significant lack-of-fit, and with very satisfactory values of *R*^2^. The latter was estimated to be 98.39, 89.26, 86.41, 97.98, and 97.98% for the TPC, TFC, TAC, anti-*S. aureus*, and anti-*S. enterica* activities, respectively. As shown in Table 2, with no lack-of-fit, *p* values of the total model of all responses were less than 0.05. Besides, *F* values from all regression models (*F*_[TPC]_ = 33.87, *p*=0.001; *F*_[TFC]_ = 4.62, *p*=0.043; *F*_[TAC]_ = 3.53, *p*=0.049; *F*_[anti-*S. aureus*]_ = 26.88, *p*=0.001 and *F*_[anti-*S. enterica*]_ = 26.13, *p*=0.001) were significant. Second-order polynomial equations were used to study the relation between the input process variables (*X*1, *X*2, and *X*3) and their five respective responses. So, the second-order polynomial coefficient for each term of the equation was determined through multiple regression analysis using RSM.

#### 3.1.1. Effect of Extraction Variables on Phytochemical Contents

*(1) TPC*. As shown in Table 1, the TPCs of different experimental runs ranged between 70.2 and 123.8 mg GAE/g. The data obtained from the experiments were used to estimate the coefficients by regression analysis. *p* values were used to evaluate the significance of different coefficients, which provides the information required to understand the interaction patterns among the experimental variables. *p*-values less than 0.05 are always accepted as the values for statistical significance with a confidence level greater than 95%, and smaller *p* values refer to a larger significance of the respective coefficient. As presented in Table 2, the linear (*p*=0.002), the quadratic (*p* ≤ 0.001), and interaction (*p*=0.018) coefficients were significant. The response of activity can be expressed in the following regression equation:

(4) $$\begin{array}{c} Y_{\text{TPC}}=70-9.5\times X1-3.312\times X2-8.137\times X3+29.9\times X1^{2}+11.225\times X2^{2}+9.025\times X3^{2}+2.175\times X1X2+0.875\times X1X3+10.95\times X2X3. \end{array}$$

**Figure 1:**
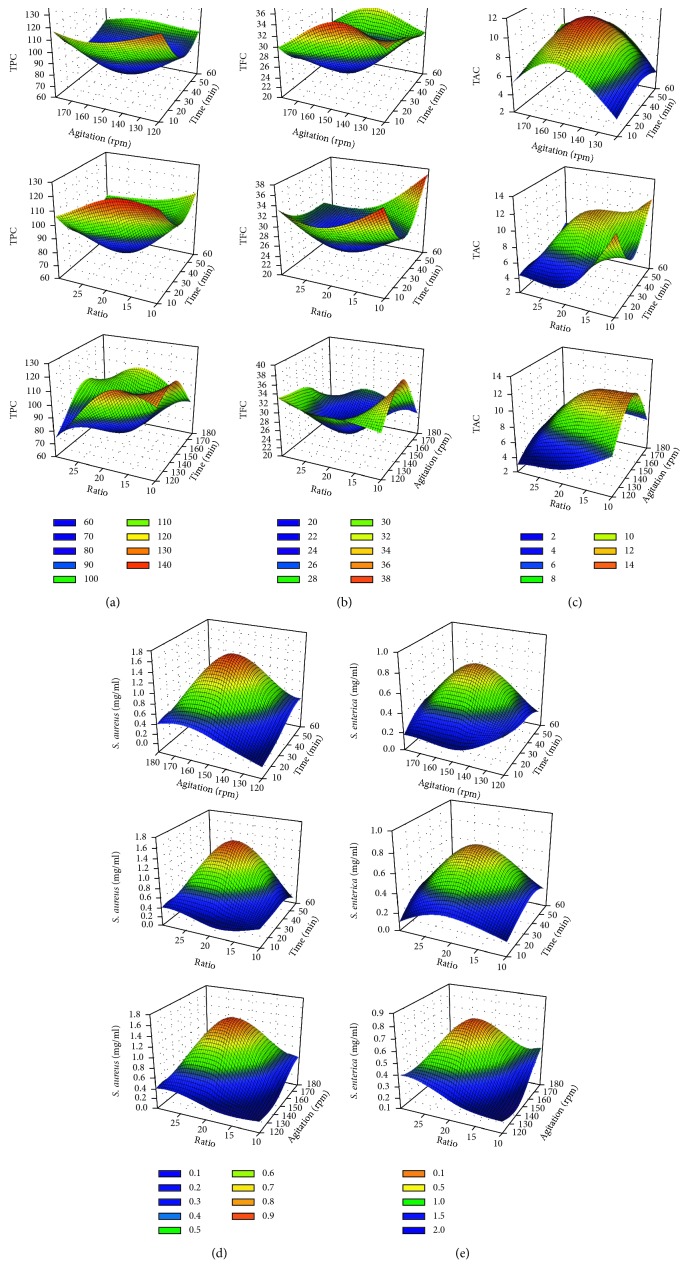
Three-dimensional presentation of the developed response surface model for the TPC (a), TFC (b), TAC (c), anti-*S. aureus* (d), and anti-*S. enterica* (e) activities from pomegranate peel with respect to extraction time, agitation speed, and solvent/solid ratio.

The quadratic term coefficients (*X*1^2^, *X*2^2^, and *X*3^2^) and the interaction coefficients (*X*1*X*2, *X*1*X*3, and *X*2*X*3) have a positive effect while *X*1, *X*2, and *X*3 exhibit a negative effect on the TPC. A longer extraction time presents a negative effect on the TPC. Meanwhile, no influence of this parameter was found, indicating that there is no benefit in using contact times greater than 15 min. Similar effects of time (10–30 min) on TPC pomegranate peel extracted by ultrasonic procedure was observed [33]. On the same line, when the solvent/solid ratio increased from 10 to 30, the extraction efficiency of the TPC did not vary significantly. Dailey et al. in 2015 and Medouni-Adrar et al. in 2015 found that the extraction time and sample-to-solvent ratios have respectively a negative effect on the Macadamia (*Macadamia tetraphylla*) skin waste and the Algerian skin grape TPCs [50, 51]. The 3-D response surface plot of the regression equation (4) was presented in Figure 1(a). From the graphical representation, it can be concluded that the TPC does not depend on the agitation speed and extraction time. In this regard, the TPC increased with the increase of the solvent/solid ratio from 12 to 18, and then slightly decreased from 19 to 23. With a minimal value of extraction time, the maximum of TPC in the PPE was observed. Likewise, Dailey and Vuong observed that TPC did not change significantly by extraction time (10–30 min) of *Macadamia tetraphylla* [50]. However, Pinelo et al. showed that the TPC of grape extracts decrease with the increase of the extraction time (30–90 min) [52].

**Figure 2:**
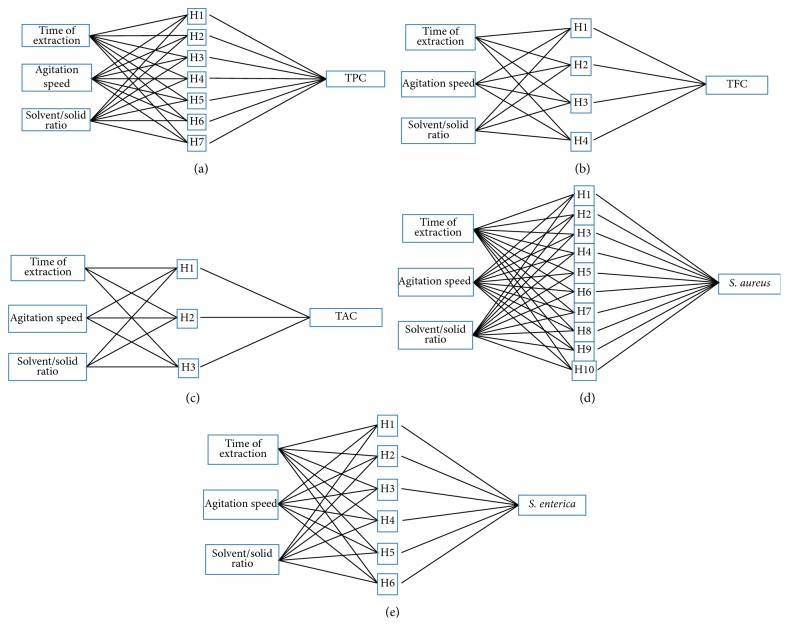
Graphical representation of the MLP neural network of the TPC (a), TFC (b), TAC (c), anti-*S. aureus* (d), and anti-*S. enterica* (e) activities with respect to the extraction time, agitation speed, and solvent/solid ratio.

Based on numerical optimization, the predicted models with the maximum TPC was 124.5 mg GAE/g, when the optimal values of test factors were: time of extraction (10 min), agitation speed (135 rpm) and solvent/solid ratio (15) (Table 1, Figure 2(a)). In fact, an increase of 56.224% was shown between the original (70 mg GAE/g) and the optimized TPC (124.5 mg GAE/g).

*(2) TFC*. The maximum and minimum TFCs were 37.11 and 22.00 mg QE/g, respectively (Table 1). The quadratic models (*X*1^2^, *X*2^2^, and *X*3^2^) have a positive effect on TFC while linear and interaction models of time of extraction and solvent/solid ratio have negative effects. The generalized second-order polynomial equation proposed for TFC is as follows:

(5) $$\begin{array}{c} Y_{\text{TFC}}=23.037-1.731\times X1-2.34\times X2-1.016\times X3+6.666\times X1^{2}+0.384\times X2^{2}+4.67\times X3^{2}+0.588\times X1X2-1.875\times X1X3-1.092\times X2X3. \end{array}$$

Figure 1(b) shows the influence of the three studied parameters on the TFC. In this regard, the increase of agitation speed from 140 rpm to 150 rpm increased the TFC. The increase of extraction time and solvent/solid ratio has a negative effect on the TFC (Figure 1(b)). Dailey and Vuong has demonstrated the negative interaction between the time and ratio on TFC [50]. On the contrary, Sood and Gupta observed that the interaction ((ethanol/pomegranate powder ratio) and (ethanol/extraction time)) has a positive effect on the TFC [34].

Moreover, greater extraction time (*X*1), having a negative impact on the TPC and TFC, increase the chances of free radicals formation which can be scavenged by phenolic compounds [53, 54]. Equally, plant cells contain enzymes, in particular polyphenol oxidase, capable of altering the phenolic compounds and contribute to enzymatic browning reactions [55]. Furthermore, excessive time is not useful to extract more phenolic compounds [56, 57]. The optimization allowed attaining a maximum TFC of 36.292 mg QE/g with a 63.47% of increase compared with standard conditions. To get this TFC, the time of extraction was set at 8.5 min, agitation speed at 132 rpm, and solvent/solid ratio at 11.25.

*(3) TAC*. As shown in Table 2, the interaction effect was the most significant (*F*-value of 5.42, *p*=0.031), followed by the linear effects (*F* values of 4.25, *p*=0.048).

The regression equation showing the mathematical relationship of process variables for the TAC is as follows:

(6) $$\begin{array}{c} Y_{\text{TAC}}=4.633+1.546\times X1+2.435\times X2-0.572\times X3+0.731\times X1^{2}+1.147\times X2^{2}+2.872\times X3^{2}+0.753\times X1X2-1.192\times X1X3+2.276\times X2X3. \end{array}$$

Figure 1(c) showed that the increment in agitation speed had both positive and negative (or dual) effects on the TAC. More precisely, enhancement of the intensity level of the agitation speed up to 130 rpm led to a negligible increase in the TAC. By a further increment of the intensity level >160 rpm, the TAC was decreased. In a fixed extraction time, the TAC can also be increased by the increasing the solvent/solid ratio up to 15.5 (Figure 1(c)).

The optimization allowed as well a maximum TAC of 7.182 mg cy-3-glu/100 g under the following conditions: time of extraction of 35 min, agitation speed of 155 rpm, and solvent/solid ratio of 17. These conditions contributed to a 64.6% increase of TAC in comparison with standard conditions.

#### 3.1.2. Effect of Extraction Variables on the Antibacterial Activity

*(1) Anti-S. aureus activity*. The anti-*S. aureus* activity (MIC, *Y*_anti-*S. aureus*_) of PPE, varied from 0.097 to 1.56 mg/ml, was significantly (*p* < 0.05) affected by the quadratic effects (*F* values of 78.07, *p* ≤ 0.001) (Tables 1 and 2). The response of anti-*S. aureus* activity can be expressed by the following regression equation:

(7) $$\begin{array}{c} Y_{\text{anti}-S\text{. aureus}}=1.56-0.033\times X1+0.038\times X2-0.014\times X3-0.596\times X1^{2}-0.519\times X2^{2}-0.696\times X3^{2}+0.025\times X1X2+0.024\times X1X3-0.15\times X2X3. \end{array}$$

Equation (7) showed that *X*2 and the interaction coefficients (*X*1*X*2 and *X*1*X*3) have a positive effect, while, quadratic term coefficients and the single effect of *X*1 and *X*3 showed a negative effect on the MIC_anti-*S. aureus*_ activity. The 3D response surfaces based on equation (7) was presented in Figure 1(d): the dark violet color regions in each response surface plot represent the regions where maximum anti–*S. aureus* activity was observed. At a high agitation speed (180 rpm), and by increasing the extraction time, MIC _anti-*S. aureus*_ activity was decreased with a maximum at a level ranged between 15 and 20 min. The interaction effect *X*1*X*3 showed that the anti-*S. aureus* activity goes to a maximum activity at 0.2 mg/ml at a high value of extraction time (60 min) and at a minimum level of ratio. While, minimum anti-*S. aureus* activity was obtained at the middle levels of extraction time (30–40 min), agitation speed (145–155 rpm), and solvent/solid ratio (17–22). At optimized conditions extraction time at 11 min, agitation speed 125 rpm and solvent/solid ratio 12, anti-*S*. *aureus* activity remarkably decreased from 1.56 to 0.171 mg/mL.

PPE is widely recognized for its effectiveness against a broad spectrum of bacterial pathogens. Our results are in the same line with the obtained results reported by Dahham et al. and Ismail et al. who studied the antimicrobial effect of PPE against *S. aureus* [58, 59].

*(2) Anti-S. enterica activity*. Regression analysis data such as estimated coefficients and *p*-value were shown in Table 2, and the response of anti-*S. enterica* activity was predicted by the following regression equation:

(8) $$\begin{array}{c} Y_{\text{anti}-S\text{. enterica}}=0.78+0.011\times X1+0.008\times X2+0.003\times X3-0.341\times X1^{2}-0.188\times X2^{2}-0.263\times X3^{2}+0.095\times X1X2+0.027\times X1X3-0.091\times X2X3. \end{array}$$

All linear and interactions (*X*1*X*2 and *X*1*X*3) exhibit a positive effect on anti-*S. enterica* activity, while quadratic term coefficients (*X*1^2^, *X*2^2^, and *X*3^2^) and interaction *X*2*X*3 present a negative effect. At a constant ratio of solvent to solid (*X*3), the increasing of extraction time (*X*1) to 60 min and a minimal speed agitation (*X*2) to 120 rpm increases the anti-*S. enterica* activity (Figure 1(e)). The best conditions to produce the minimal MIC against *S. enterica*, at 0.555 mg/ml, were resulted in the following responses: time of extraction, 12 min; agitation speed, 129.675 rpm, and solvent/solid ratio; 22.5. These conditions contributed to an increase of 71.19 % in MIC_anti-*S. enterica*_ activity in comparison with standard conditions.

Wafa et al. reported that MICs values of pomegranate ethanolic extract against *Salmonella* strains ranged between 10.75 and 12.5 mg/mL [19]. Results reported by Choi et al. showed that the MIC values of PPE against *Salmonella* strains ranged from 0.0625 to 1 mg/mL [60]. Among the tested *Salmonella* strains, *Salmonella typhimurium* and *Salmonella anatum* were found to be more sensitive with an MIC value of 0.25 mg/mL [61]. Different inhibition profile of PPE against microorganisms could be in part due to the different extraction solvents, the plant variety, variations in the geographical, and the climatic conditions [60].

### 3.2. Artificial Neural Network Prediction on Phytochemical Contents and Antibacterial Activity

ANN has been widely used as a cutting-edge tool for the simulation and optimization of bioactive compounds extraction from different matrices [62, 63]. In this study, ANN was used in combination with an experimental design in order to obtain maximum phytochemical contents with the highest antibacterial activity.  Figure 2 showed the structure of the multilayer perceptron ANN model with a back-propagation learning algorithm, designed for the relevant process. The first step of ANN modelling was the optimization of a neural network with the aim of obtaining an ANN model with minimal dimension and minimal errors in training and testing. The design of experiments and their respective experimental yields were used for training the network. The input layer represents vectors constituted of noncoded variables: time of extraction, agitation speed, and solvent/solid ratio, and the output layer represents TPC, TFC, TAC, anti-*S. aureus*, and anti-*S. enterica* activities. In the ANN structure, the unit number in the hidden layer was set as 7, 4, 3, 10, and 6 for the node number [64, 65]. For TPC, the most suitable model is an MLP trained with 3 independent variables for the input parameters (3-7-1): 3 input neurons, 7 hidden neurons, and 1 output (Figure 2(a)). The results of the trained model were created with high *R*^2^ (*R*^2^ = 99.88% for test). The topology of the ANN architecture illustrated in Figures 2(b)–2(c), showed a neural structure of (3-4-1) and (3-3-1) respectively for TFC and TAC with *R*^*2*^ = 99.58 and 99.21%, respectively. For anti-*S. aureus* and *S. enterica* activities, the robustness network architecture with (3-10-1) and (3-6-1), respectively, were represented in Figures 2(d)–2(e). Furthermore, given *R*^*2*^ were 99.13 and 99.67%, which is totally satisfactory.

**Figure 3:**
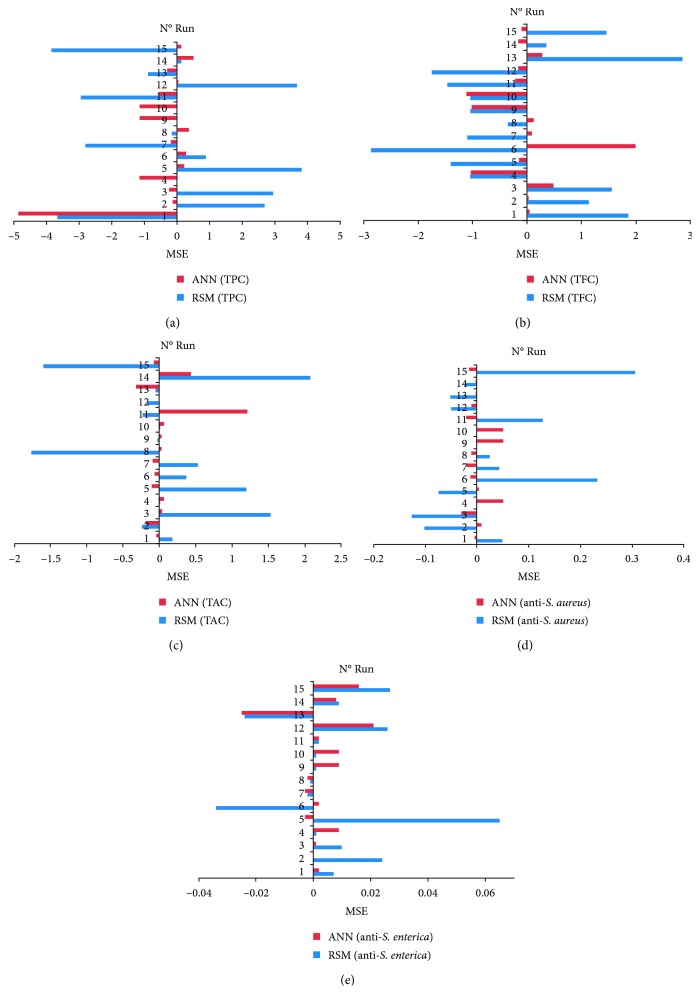
Comparison of the predictive capacity of RSM and ANN for five model outputs.

The predicted data by ANN model were given along with the RSM predicted and experimental values (Table 1, Figure 3). In fact, for predicted ANN, the relative percent deviation between the phytochemical contents and the antibacterial activities obtained experimentally for the same extraction conditions were respectively: 1.005, 1.003, 1.004, 1.031, and 1.005% for the TPC, TFC, TAC, anti-*S. aureus*, and anti-*S. enterica* activities.

### 3.3. Comparison of RSM and ANN Models

For a comparison of both well trained ANN and RSM models, *R*^2^ and mean square error (MSE) were used as statistical indicators (Table 1, Figure 3).

(9) $$\begin{array}{c} \text{MSE}=\frac{1}{n}\overset{n}{\underset{i-1}{\sum }}\left(Y_{\exp.}-Y_{\text{pred}.}\right). \end{array}$$

Hence, for phytochemical contents and antibacterial activities, obtained results from RSM models indicated that the measured *R*^2^ between the observed and the predicted data were acceptable with *R*^2^ > 70%. Furthermore, ANN models showed higher accuracies when compared with RSM models. The comparison between results with predicted outputs from ANN and RSM was shown in Figure 3. ANN model improved the adjustments in comparison with the RSM model. ANN model presents an improvement of 4.4, 26.66, 28, 4.8 and 5.5% in terms of *R*^2^ concerning the TPC, TFC, TAC, anti-*S. aureus*, and anti-*S. enterica* activities (Figure 3). To illustrate the difference between the mentioned variables, enlarged versions of the simulation output based on the ANN and RSM models are presented in Figure 3. The predictive superiority of the ANN model over the RSM model for data fitting and the estimation capability has already been demonstrated in previous studies [64–68].

### 3.4. Prediction of the Antibacterial Activity of PPE

To elucidate a better understanding of the PPE as an antibacterial agent, the linear (ANOVA) and general (ANCOVA) models were mathematically used to model the growth rate of *S. aureus* ATCC 6538 and *S. enterica* subsp. *enterica* serovar Typhimurium ATCC 14028. Challenge experiments were conducted to evaluate PPE effects on the growth *S. aureus* and *S. enteric*, respectively, in Chapman and XLD media. Therefore, *S. aureus* and *S. enterica* kinetics were performed using different concentrations of PPE (MIC, 2 × MIC, 4 × MIC, and 8 × MIC). To investigate the effects of PPE, *S. aureus* and *S. enterica* growth were followed during 26 h and evaluated with respect to the control (culture without PPE addition). Moreover, it should be noted that the PPE addition to bacterial cells was realized after 3 hours of incubation when growth reached the beginning of the exponential phase (Cell∼10^4^ CFU/ml).

Viable cell counts log_10_ (CFU/ml) in absence Control (0MIC) and in presence of 1 × MIC, 2 × MIC, 4 × MI and 8 × MIC of pomegranate peel extracts. The time of extracts addition was 3 hours. ±: Standard deviation of three replicates; Values with a different letter (a–e) within a row of the same time are significantly different (*p* < 0.05); Values with a different letter (A–L) within a column of the same concentration are significantly different (*p* < 0.05).

#### 3.4.1. Influence of the PPE on In Vitro *S. aureus* and *S. enterica* Inhibition Using Linear Model (ANOVA)

A rapid killing action occurred at 1 hour after addition of all tested MICs (incubation time of 4 hours). For *S. aureus*, these population numbers were 0.74, 1.02, 1.32, and 1.85 log_10_ CFU/mL lower (*p* < 0.05) than the control numbers. Meanwhile, for *S. enterica*, 1 h post PPE addition, at all tested MICs, PPE showed a reduction (*p* < 0.05) of 0.24, 0.4, 0.71, and 1.15 log_10_ CFU/mL compared with control samples (Table 3). At 4 × MIC, no viable cells were observed after 13 and 11h respectively for *S. aureus* and *S. entreica*. Interestingly, at 8 × CMI, the early logarithmic growth phase of *S. aureus* ATCC 6538 resulted in a rapid decrease during 5 hours, while 7 hours were enough to inhibit *S. enterica* ATCC 14028 (Table 3). These results clearly demonstrated that PPE has bactericidal activity against the Gram-positive (*S. aureus* ATCC 6538) and the Gram-negative (*S. enterica* ATCC 14028) bacteria.

Table 4 shows the overall differences between 5 trials for each bacterium at all tested MICs (1 × MIC, 2 × MIC, 4 × MIC and 8 × MIC) and 20 different times. For one fixed time, significant differences (*p* < 0.001) were observed between treatments trials and times (Trial × Time). The five treatment groups significantly increased the anti-*S. aureus* (*p* < 0.001) and anti-*S. enterica* (*p* < 0.001) activities. Indeed, this result confirmed the previous results showed in Table 3 where PPE exerts dose-dependent bactericidal effects.

#### 3.4.2. Influence of the PPE on In Vitro *S. aureus* and *S. enterica* Inhibition Using General Linear Model (ANCOVA)

A descriptive statistics using a mixed model of the time-related survival of *S. aureus* and *S. enterica* following treatment with various PPE concentrations was presented in Table 5. *p*-value < 0.05 was always accepted value for statistical significance with confidence level >95% and smaller *p* values refer to a larger significance of the respective coefficient [69, 70]. As shown in Table 5, for *S. aureus*, a significant effect (*p* < 0.05) was noticed at 0, 1, 2, 3, 4, and 5 hours. In fact, in the beginning of experimentation, the first 2 hours after PPE addition were primordial for *S. aureus* inhibition. Concerning *S. enterica* inhibition, 3 hours of post PPE addition was effective. In contrast, no significant differences (*p* > 0.05) were found between groups at any time point 2 and 3 hours post PPE addition, respectively, on *S. aureus* and *S. enterica* inhibition (Table 5).

The effects of treatments, time, and their interaction on the inhibition of *S. aureus* and *S. enterica* were shown in Table 6. A significant interaction (*p* < 0.05) between all treatments samples and bacterial growth time was remarked. With the lowest *p* value, 8 × MICs (Trial 5), and incubation was found highly significant (*p* < 0.01) effect regarding inhibition of *S. aureus* and *S. enterica* (Table 6). It is obvious that high concentrations of 1.368 mg/mL against *S. aureus*) and (4.44 mg/mL against *S. enterica*) coupled with time have an active action on inhibition of studied bacteria.

The covariance parameters are presented in Table 7(A). Intercept variances are estimated as 5.379 and 1.788 for *S. aureus* and *S. enterica*, respectively. The null hypothesis for this parameter is a variance of zero, which would indicate that a random effect is not needed, and the statistical test is called a Wald Z statistic [71]. For *S. aureus* and *S. enterica*, the two hypotheses Wald *Z* = 0.000 where *p*=0.015; for anti-*S. aureus* activity and *p*=0.018; for anti-*S. enterica* activity was accepted (Table 7(A)). Equally, as illustrated in Table 7(B) for fixed time, the two hypotheses Wald *Z* = 0.815, *p*=0.038 and Wald *Z* = 0.000, *p*=0.031 as well as Wald *Z* = 0.000, *p*=0.029 were accepted. Furthermore, results from Table 7 showed that interactions of the trial type and incubation time have an important role in inhibition of *S. aureus* and *S. enterica*.

## 4. Conclusion

Recent years have witnessed an indisputable interest in plants for exploring and promoting their bioactive compounds in the health field. In this study, the use of pomegranate peel as a natural alternative source to produce biological compounds, phytochemical contents (TPC, TFC, and TAC), and anti-*S. aureus* and anti-*S. enterica* activities, has been evaluated and optimized. Both RSM and ANN have been used to generate the model on all five responses from PPE. The extraction time, agitation speed, and solvent/solid ratio of pomegranate peel markedly influenced phytochemical contents and antibacterial activity. Under optimal conditions, TPC (124.5 mg GAE/g), TFC (36.292 mg QE/g), TAC (7.182 mg cy-3-glu/100 g), MIC_anti-*S. aureus*_ (0.171 mg/mL), and MIC_anti-*S. enterica*_ (0.555 mg/mL) were maximal. Equally, this work highlights the great anti-*S. aureus* and anti-*S. enterica* effects of PPE at optimized conditions. The mode of action study confirms our results and indicates that PPE exerts a dose-dependent bactericidal effect against these foodborne bacterial pathogens. Therefore, PPE can be considered as a strong and promising tool for future application as a safe method for the preservation of food products.

## Figures and Tables

**Table 1:** Box–Behnken design and ANN models of variables (in coded levels), with phytochemical contents (TPC, TFC, and TAC) and anti-*S. aureus* and *S.enterica* activity responses.

Run order	(*X*1)	(*X*2)	(*X*3)	Recovery extraction (%)	Phytochemical content									Antibacterial activity					
					TPC (mg GAE/g)			TFC (mg QE/g)			TAC (mg cy-3-glu/100 g)			*S. aureus* (MIC mg/ml)			*S. enterica* (MIC mg/ml)		
					Exp.	Pred. RSM	Pred. ANN	Exp.	Pred. RSM	Pred. ANN	Exp.	Pred. RSM	Pred. ANN	Exp.	Pred. RSM	Pred. ANN	Exp.	Pred. RSM	Pred. ANN
1	10 (−1)	150 (0)	10 (−1)	18.45 ± 0.92^c^	123.8 ± 6.19^cd^	127.485	128.671	37.11 ± 1.85^f^	35.249	37.06	4.78 ± 0.22^c^	4.607	4.817	0.39 ± 0.00^b^	0.34	0.395	0.195 ± 0.00^c^	0.188	0.193
2	10 (−1)	180 (+1)	20 (0)	19.98 ± 0.95^d^	118.02 ± 5.91^c^	115.312	118.15	30.02 ± 1.45^d^	28.892	30.006	4.64 ± 0.21^c^	4.884	4.828	0.39 ± 0.00^b^	0.492	0.382	0.195 ± 0.00^c^	0.171	0.195
3	60 (+1)	150 (0)	10 (−1)	17.3 ± 0.86^b^	109.6 ± 4.48^bc^	106.637	109.849	37.09 ± 1.84^f^	35.535	36.606	11.61 ± 0.52^h^	10.085	11.582	0.097 ± 0.00^a^	0.224	0.128	0.195 ± 0.00^c^	0.185	0.194
4	35 (0)	150 (0)	20 (0)	17 ± 0.85^b^	70.02 ± 3.49^a^	70.00	71.156	22.00 ± 1.22^a^	23.037	23.019	4.64 ± 0.21^c^	4.633	4.586	1.56 ± 0.00^d^	1.56	1.509	0.781 ± 0.00^b^	0.780	0.772
5	10 (−1)	120 (−1)	20 (0)	15.6 ± 0.7^ab^	130 ± 6.47^d^	126.162	129.789	33.35 ± 1.62^de^	34.749	33.507	3.71 ± 0.17^b^	2.520	3.81	0.39 ± 0.00^b^	0.464	0.386	0.39 ± 0.00^a^	0.325	0.393
6	35 (0)	180 (+1)	10 (−1)	15 ± 0.75^a^	85.00 ± 4.23^ab^	84.125	84.729	25.00 ± 1.22^b^	27.862	23.019	9.75 ± 0.46^g^	9.385	9.808	0.781 ± 0.00^c^	0.547	0.794	0.39 ± 0.00^a^	0.424	0.388
7	60 (+1)	120 (−1)	20 (0)	15.15 ± 0.6^a^	99.91 ± 4.92^b^	102.712	100.108	29.00 ± 1.44^cd^	30.108	28.917	3.94 ± 0.12^b^	3.406	4.033	0.39 ± 0.00^b^	0.347	0.411	0.195 ± 0.00^c^	0.197	0.198
8	35 (0)	120 (−1)	10 (−1)	16.7 ± 0.45^b^	112.5 ± 5.61^bc^	112.65	112.16	30.01 ± 1.5^d^	30.357	29.897	7.01 ± 0.33^f^	8.766	6.982	0.195 ± 0.00^e^	0.170	0.206	0.195 ± 0.00^c^	0.196	0.197
9	35 (0)	150 (0)	20 (0)	14.4 ± 0.51^a^	70.00 ± 3.41^a^	70.00	71.156	22.01 ± 1.02^a^	23.037	23.019	4.61 ± 0.21^c^	4.633	4.586	1.56 ± 0.00^d^	1.56	1.509	0.781 ± 0.00^b^	0.780	0.772
10	35 (0)	150 (0)	20 (0)	17.85 ± 0.82^bc^	70.01 ± 3.33^a^	70.00	71.156	22.00 ± 1.09^a^	23.037	23.111	4.64 ± 0.22^c^	4.633	4.586	1.56 ± 0.00^d^	1.56	1.509	0.781 ± 0.00^b^	0.780	0.772
11	10 (−1)	150 (0)	30 (+1)	17.05 ± 0.79^b^	106.5 ± 5.25^bc^	109.462	107.043	35.50 ± 1.75^e^	36.965	35.706	5.32 ± 0.20^d^	5.547	4.112	0.39 ± 0.00^b^	0.263	0.411	0.195 ± 0.00^c^	0.193	0.193
12	60 (+1)	150 (0)	30 (+1)	15 ± 0.66^a^	95.8 ± 4.77^b^	92.112	95.755	28.00 ± 1.39^c^	29.751	28.151	6.38 ± 0.30^e^	6.554	6.384	0.195 ± 0.00^e^	0.245	0.206	0.39 ± 0.00^a^	0.364	0.369
13	35 (0)	120 (−1)	30 (+1)	17.27 ± 0.84^b^	73.6 ± 3.66^a^	74.475	73.895	33.36 ± 1.66^de^	30.508	33.071	3.02 ± 0.14^a^	3.068	3.341	0.39 ± 0.00^b^	0.442	0.391	0.39 ± 0.00^a^	0.414	0.415
14	35 (0)	180 (+1)	30 (+1)	17.35 ± 0.36^b^	89.9 ± 4.44^ab^	89.75	89.397	24.00 ± 1.15^b^	23.643	24.154	14.86 ± 0.71^i^	12.792	14.434	0.195 ± 0.00^e^	0.219	0.197	0.39 ± 0.00^a^	0.381	0.382
15	60 (+1)	180 (+1)	20 (0)	17 ± 0.49^b^	96.6 ± 4.81^b^	100.437	96.482	28.06 ± 1.32^c^	26.605	28.151	7.89 ± 0.37^f^	9.484	7.961	0.781 ± 0.00^c^	0.475	0.797	0.39 ± 0.00^a^	0.363	0.374

Variables: extraction time (*X*1), agitation speed (*X*2), and solvent/solid ratio (*X*3). ±: Standard deviation of three replicates; Averages with different letters (a–i) in the same column, for each parameter, are different (*p* < 0.05).

**Table 2:** ANOVA results of process variables against TPC, TFC, TAC, anti-*S. aureus*, and anti-*S. enterica* activity responses.

Responses	Regression	Sum of square	*F* value	*p* value	*R* ^2^	*R*_adjusted_^2^
TPC	Linear	1347.15	24.51	0.002^*∗*^	98.39	95.48
	Quadratic	3735.17	67.96	0.000^*∗∗*^		
	Interaction	501.60	9.13	0.018^*∗*^		
	Total model	5583.92	33.87	0.001^*∗∗*^		

TFC	Linear	76.072	3.24	0.079	89.26	72.92
	Quadratic	228.990	9.75	0.016^*∗*^		
	Interaction	20.225	0.86	0.119		
	Total model	325.287	4.62	0.043^*∗*^		

TAC	Linear	69.208	4.25	0.048^*∗*^	86.41	71.95
	Quadratic	37.531	2.94	0.078		
	Interaction	28.690	5.42	0.031^*∗*^		
	Total model	135.429	3.53	0.049^*∗*^		

*S. aureus*	Linear	0.022	2.18	0.153	97.98	94.33
	Quadratic	3.561	78.07	0.000^*∗∗*^		
	Interaction	0.094	2.08	0.222		
	Total model	3.678	26.88	0.001^*∗∗*^		

*S. enterica*	Linear	0.001	1.77	0.215	97.92	94.17
	Quadratic	0.718	71.08	0.000^*∗∗*^		
	Interaction	0.072	7.17	0.029^*∗*^		
	Total model	0.809	26.13	0.001^*∗∗*^		

^*∗*^ *p*=0.05; ^*∗∗*^*p*=0.001; ^*∗∗∗*^*p* < 0.001.

**Table 3:** Influence of the dose of PPE addition on the growth of *Staphylococcus aureus* ATCC 6538 and *Salmonella enterica* subsp. *enterica* serovar Typhimurium ATCC 14028 *in vitro* using linear model (ANOVA).

*Staphylococcus aureus* ATCC 6538						*Salmonella enterica* subsp. *enterica* serovar typhimurium ATCC 14028					
Time (h)	0 × MIC	1 × MIC	2 × MIC	4 × MIC	8 × MIC	Time (h)	0 × MIC	1 × MIC	2 × MIC	4 × MIC	8 × MIC
0	4.00 ± 0.18^aAB^	4.00 ± 0.19^aF^	4.00 ± 0.15^aH^	4.00 ± 0.17^aI^	4.00 ± 0.18^aF^	0	4.00 ± 0.16^aA^	4.00 ± 0.20^aI^	4.00 ± 0.16^aI^	4.00 ± 0.18^aH^	4.00 ± 0.19^aG^
1	4.35 ± 0.21^aA^	4.35 ± 0.20^aGH^	4.35 ± 0.19^aHI^	4.35 ± 0.20^aJ^	4.35 ± 0.21^aGH^	1	4.25 ± 0.17^aAB^	4.25 ± 0.18^aJ^	4.25 ± 0.20^aJ^	4.25 ± 0.17^aI^	4.25 ± 0.20^aH^
2	4.80 ± 0.22^aAB^	4.80 ± 0.24^aHI^	4.80 ± 0.22^aJ^	4.80 ± 0.19^aK^	4.80 ± 0.18^aHI^	2	4.50 ± 0.21^aB^	4.50 ± 0.22^aK^	4.50 ± 0.19^aK^	4.50 ± 0.21^aK^	4.50 ± 0.22^aI^
3	5.02 ± 0.24^aAB^	5.02 ± 0.22^aI^	5.02 ± 0.25^aK^	5.02 ± 0.20^aL^	5.02 ± 0.23^aI^	3	4.93 ± 0.24^aC^	4.93 ± 0.23^aL^	4.93 ± 0.24^aM^	4.93 ± 0.21^aL^	4.93 ± 0.24^aJ^
4	5.34 ± 0.25^eAB^	4.63 ± 0.22^dH^	4.35 ± 0.19^cI^	4.05 ± 0.19^bI^	3.52 ± 0.16^aE^	4	5.15 ± 0.24^dC^	4.91 ± 0.22^cdL^	4.75 ± 0.22^cL^	4.44 ± 0.21^bK^	4.00 ± 0.19^aG^
5	6.57 ± 0.32^eB^	4.60 ± 0.22^dH^	4.20 ± 0.20^cHI^	3.81 ± 0.180^bH^	3.01 ± 0.14^aD^	5	5.80 ± 0.27^dD^	4.80 ± 0.23^cL^	4.54 ± 0.21^bcK^	4.21 ± 0.00^bI^	3.22 ± 0.00^aF^
6	7.09 ± 0.34^eBC^	4.50 ± 0.21^dH^	4.15 ± 0.19^cH^	3.70 ± 0.17^bH^	2.51 ± 0.12^aC^	6	6.02 ± 0.28^dE^	4.50 ± 0.21^cK^	4.22 ± 0.20^bcIJ^	4.00 ± 0.18^bH^	2.55 ± 0.12^aE^
7	8.25 ± 0.39^eBC^	4.24 ± 0.18^dG^	3.60 ± 0.17^cG^	3.22 ± 0.15^bG^	1.89 ± 0.09^aB^	7	6.15 ± 0.29^dEF^	4.30 ± 0.19^cJ^	3.81 ± 0.18^bH^	3.58 ± 0.17^bG^	2.00 ± 0.08^aD^
8	8.52 ± 0.16^eBC^	3.91 ± 0.18^dF^	3.44 ± 0.16^cF^	3.00 ± 0.14^bF^	1.00 ± 0.00^aA^	8	6.25 ± 0.29^eF^	4.00 ± 0.19^dI^	3.45 ± 0.16^cG^	3.00 ± 0.12^bF^	1.75 ± 0.07^aC^
9	9.02 ± 0.43^eC^	3.65 ± 0.17^dE^	3.00 ± 0.13^cE^	2.61 ± 0.12^bE^	1.00 ± 0.00^aA^	9	6.45 ± 0.30^dFG^	3.51 ± 0.16^cH^	3.33 ± 0.15^cG^	2.54 ± 0.12^bE^	1.33 ± 0.06^aB^
10	9.32 ± 0.44^dCD^	3.11 ± 0.14^cD^	2.74 ± 0.13^bD^	2.42 ± 0.11^bD^	1.00 ± 0.00^aA^	10	6.55 ± 0.31^dG^	3.26 ± 0.15^cG^	3.00 ± 0.14^cF^	2.11 ± 0.10^bD^	1.00 ± 0.00^aA^
11	9.56 ± 0.45^eCD^	2.77 ± 0.13^dC^	2.41 ± 0.11^cC^	2.00 ± 0.09^bC^	1.00 ± 0.00^aA^	11	6.74 ± 0.32^eGH^	3.04 ± 0.15^dF^	2.71 ± 0.13^cE^	1.75 ± 0.08^bC^	1.00 ± 0.00^aA^
12	9.80 ± 0.47^eCD^	2.40 ± 0.11^dBC^	1.90 ± 0.09^cB^	1.55 ± 0.07^bB^	1.00 ± 0.00^aA^	12	6.85 ± 0.33^eH^	2.75 ± 0.14^dE^	2.42 ± 0.11^cD^	1.44 ± 0.06^bB^	1.00 ± 0.00^aA^
14	10.52 ± 0.50^dD^	2.35 ± 0.11^cBC^	1.44 ± 0.07^bAB^	1.22 ± 0.05^abAB^	1.00 ± 0.00^aA^	14	6.98 ± 0.33^dH^	2.22 ± 0.10^cD^	1.90 ± 0.09^bC^	1.00 ± 0.00^aA^	1.00 ± 0.00^aA^
16	10.77 ± 0.51^dCD^	2.25 ± 0.10^cB^	1.20 ± 0.05^bAB^	1.00 ± 0.00^aA^	1.00 ± 0.00^aA^	16	7.02 ± 0.30^dH^	1.75 ± 0.07^cC^	1.51 ± 0.07^bB^	1.00 ± 0.00^aA^	1.00 ± 0.00^aA^
18	11.02 ± 0.52^cCD^	2.14 ± 0.10^bAB^	1.10 ± 0.03^aA^	1.00 ± 0.00^aA^	1.00 ± 0.00^aA^	18	7.12 ± 0.29^dHI^	1.66 ± 0.07^cBC^	1.25 ± 0.06^bAB^	1.00 ± 0.00^aA^	1.00 ± 0.00^aA^
20	11.25 ± 0.50^cD^	2.00 ± 0.07^bAB^	1.00 ± 0.00^aA^	1.00 ± 0.00^aA^	1.00 ± 0.00^aA^	20	7.25 ± 0.33^cHI^	1.44 ± 0.06^bB^	1.00 ± 0.00^aA^	1.00 ± 0.00^aA^	1.00 ± 0.00^aA^
22	11.36 ± 0.54^cD^	1.74 ± 0.08^bAB^	1.00 ± 0.00^aA^	1.00 ± 0.00^aA^	1.00 ± 0.00^aA^	22	7.35 ± 0.33^dJ^	1.25 ± 0.05^bAB^	1.00 ± 0.00^aA^	1.00 ± 0.00^aA^	1.00 ± 0.00^aA^
24	11.50 ± 0.39^cD^	1.25 ± 0.07^bA^	1.00 ± 0.00^aA^	1.00 ± 0.00^aA^	1.00 ± 0.00^aA^	24	7.53 ± 0.34^bJ^	1.00 ± 0.00^aA^	1.00 ± 0.00^aA^	1.00 ± 0.00^aA^	1.00 ± 0.00^aA^
26	11.55 ± 0.55^cD^	1.00 ± 0.06^bA^	1.00 ± 0.00^aA^	1.00 ± 0.00^aA^	1.00 ± 0.00^aA^	26	7.55 ± 0.36^bJ^	1.00 ± 0.00^aA^	1.00 ± 0.00^aA^	1.00 ± 0.00^aA^	1.00 ± 0.00^aA^

**Table 4:** Statistics test of main and interaction effects of univariate mixed analyses of variance for all dependent variables and effects (Trial and Time) with 95% confidence intervals.

Outcome	Effect	Level number	Parameter number	*F*	df_num_/df_den_	*p* value
*Fixed trial*						
Anti-*S. aureus* activity	Intercept	1	1	206.094^*∗*^	1/60	<0.001
	Time	20	19	0.657	19/60	0.845
	Trial	1	1	66.187^*∗*^	1/60	<0.001
	Time × trial	20	19	1.291	19/60	0.224
Anti-*S. enterica* activity	Intercept	1	1	66.171	1/60	1.000
	Time	20	19	0.240	19/60	0.412
	Trial	1	1	84.122^*∗*^	1/60	<0.001
	Time × trial	20	19	1.540	19/60	0.104

*Fixed time*						
Anti-*S. aureus* activity	Intercept	1	1	1226.229^*∗*^	1/90	<0.001
	Trial	1	1	46.654^*∗*^	1/90	<0.001
	Time	5	4	3.616^*∗*^	4/90	0.009
	Trial × time	5	4	95.230^*∗*^	4/90	<0.001
Anti-*S. enterica* activity	Intercept	1	1	387.703^*∗*^	1/90	<0.001
	Trial	1	1	21.093^*∗*^	1/90	<0.001
	Time	5	4	1.228	4/90	0.305
	Trial × time	5	4	15.225^*∗*^	4/90	<0.001

df_num_/df_den_ = degrees of freedom of numerator/denominator.

**Table 5:** Analyses of covariance for all dependent variables and effect of *S. aureus* ATCC 6538 and *S. enterica* ATCC 14028 activities.

Parameter	*Staphylococcus aureus* ATCC 6538							*Salmonella enterica* subsp. *enterica* serovar typhimurium ATCC 14028						
	Estimate	SE	df	*t*	Sig.	LB	UB	Estimate	SE	df	*t*	Sig.	LB	UB
Intercept	9.44	2.432	60	3.88	0.000^*∗∗∗*^	4.573	14.306	6320	3203.043	0	1.973	1.000	−79955.35	92595.35
Hour 0	−5.44	3.44	60	−1.581	0.119	−12.321	1.441	−6316	4529.787	0	−1.394	1.000	−128327.77	115695.77
Hour 1	−5.089	3.44	60	−1.479	0.144	−11.97	1.792	−2070	4529.787	0	−0.457	1.000	−124081.77	119941.77
Hour 2	−4.64	3.44	60	−1.349	0.182	−11.521	2.241	−1820	4529.787	0	−0.402	1.000	−123831.77	120191.77
Hour 3	−4.42	3.44	60	−1.285	0.204	−11.301	2.461	−1420	4529.787	0	−0.313	1.000	−123431.77	120591.77
Hour 4	−3.859	3.44	60	−1.122	0.266	−10.74	3.022	−844	4529.787	0	−0.186	1.000	−122855.77	121167.77
Hour 5	−2.623	3.44	60	−0.763	0.449	−9.504	4.258	−89	4529.787	0	−0.020	1.000	−122100.77	121922.77
Hour 6	−2.079	3.44	60	−0.605	0.548	−8.961	4.801	166	4529.787	0	0.037	1.000	−121845.77	122177.77
Hour 7	−1.248	3.44	60	−0.363	0.718	−8.129	5.633	352	4529.787	0	0.078	1.000	−121659.77	122363.77
Hour 8	−1.018	3.44	60	−0.296	0.768	−7.9	5.863	348	4529.787	0	0.077	1.000	−121663.77	122359.77
Hour 9	−0.672	3.44	60	−0.196	0.846	−7.554	6.208	422.8	4529.787	0	0.093	1.000	−121588.97	122434.57
Hour 10	−0.51	3.44	60	−0.148	0.882	−7.392	6.37	500	4529.787	0	0.110	1.000	−121511.77	122511.77
Hour 11	−0.712	3.44	60	−0.207	0.837	−7.593	6.169	491	4529.787	0	0.108	1.000	−121520.77	122502.77
Hour 12	−0.665	3.44	60	−0.193	0.847	−7.546	6.216	511	4529.787	0	0.113	1.000	−121500.77	122522.77
Hour 14	−0.181	3.44	60	−0.053	0.958	−7.062	6.7	304	4529.787	0	0.067	1.000	−121707.77	122315.77
Hour 16	−0.029	3.44	60	−0.008	0.993	−6.91	6.852	11	4529.787	0	0.002	1.000	−122000.77	122022.77
Hour 18	0.096	3.44	60	0.028	0.978	−6.785	6.977	−44	4529.787	0	−0.010	1.000	−122055.77	121967.77
Hour 20	0.205	3.44	60	0.060	0.953	−6.676	7.086	−70	4529.787	0	−0.015	1.000	−122081.77	121941.77
Hour 22	0.183	3.44	60	0.053	0.958	−6.698	7.064	−190	4529.787	0	−0.042	1.000	−122201.77	121821.77
Hour 24	0.087	3.44	60	0.025	0.980	−6.794	6.968	−120	4529.787	0	−0.026	1.000	−122131.77	121891.77
Hour 26	0^a^	0	60	—	—	—		0^a^	0	—	—	—	—	—
Trial	−2.11	0.733	60	−2.877	0.006^*∗∗*^	−3.577	−0.642	−1330	422.878	60	−3.145	0.003^*∗∗*^	−2175.882	−484.117
Hour 0 × trial	2.11	1.037	60	2.034	0.016^*∗*^	0.035	4.184	1330	598.04	60	2.224	0.009^*∗*^	133.741	2526.258
Hour 1 × trial	2.11	1.037	60	2.034	0.022^*∗*^	0.035	4.184	1330	598.04	60	2.224	0.011^*∗*^	133.741	2526.25
Hour 2 × trial	2.11	1.037	60	2.034	0.029^*∗*^	0.035	4.184	1330	598.04	60	2.224	0.015^*∗*^	133.741	2526.258
Hour 3 × trial	2.11	1.037	60	2.034	0.034^*∗*^	0.035	4.184	1330	598.04	60	2.224	0.030^*∗*^	133.741	2526.258
Hour 4 × trial	1.691	1.037	60	1.631	0.044	−0.3835	3.766	1054	598.04	60	1.762	0.039	−142.258	2250.258
Hour 5 × trial	1.326	1.037	60	1.279	0.046	−0.7486	3.401	755	598.04	60	1.262	0.044	−441.258	1951.258
Hour 6 × trial	1.13	1.037	60	1.090	0.206	−0.9447	3.205	586	598.04	60	0.980	0..049	−610.258	1782.258
Hour 7 × trial	0.775	1.037	60	0.748	0.208	−1.299	2.85	428	598.04	60	0.716	0.077	−768.258	1624.258
Hour 8 × trial	0.574	1.037	60	0.554	0.481	−1.5	2.649	352	598.04	60	0.589	0.258	−844.258	1548.258
Hour 9 × trial	0.476	1.037	60	0.459	0.548	−1.598	2.551	256.6	598.04	60	0.429	0.369	−939.658	1452.858
Hour 10 × trial	0.376	1.037	60	0.363	0.718	−1.698	2.451	144	598.04	60	0.241	0.511	−1052.258	1340.258
Hour 11 × trial	0.358	1.037	60	0.345	0.731	−1.716	2.432	115	598.04	60	0.192	0.748	−1081.258	1311.258
Hour 12 × trial	0.275	1.037	60	0.265	0.792	−1.799	2.349	29	598.04	60	0.048	0.861	−1167.258	1225.258
Hour 14 × trial	0.111	1.037	60	0.107	0.915	−1.96	2.185	14	598.04	60	0.023	0.981	−1182.258	1210.2584
Hour 16 × trial	0.045	1.037	60	0.043	0.966	−2.029	2.119	51	598.04	60	0.085	0.932	−1145.258	1247.258
Hour 18 × trial	0.006	1.037	60	0.006	0.995	−2.068	2.08	40	598.04	60	0.067	0.947	−1156.258	1236.258
Hour 20 × trial	−0.029	1.037	60	−0.028	0.978	−2.103	2.045	30	598.04	60	0.050	0.960	−1166.258	1226.258
Hour 22 × trial	−0.029	1.037	60	−0.028	0.978	−2.103	2.045	50	598.04	60	0.084	0.934	−1146.258	1246.258
Hour 24 × trial	−0.015	1.037	60	−0.015	0.988	0–2.09	2.059	30	598.04	60	0.050	0.960	−1166.258	1226.258
Hour 26 × trial	0^a^	0	—	—	—	—	—	0^a^	0	—	—	—	—	—

SE : Standard Error; df: Degrees of freedom; *t*: the Student *t*-statistic; Sig.: the *p* value (associated with the correlation); LB : Lower bound. UB : Upper bound; Trial 1: negative control; Trial 2 : 1 × MIC. Trial 3 : 2 × MIC; Trial 4 : 4 × MIC and Trial 5 : 8 × MIC; ^*∗*^*p* < 0.05; ^*∗∗*^*p* < 0.01; ^*∗∗∗*^*p* < 0.001, ^a^This parameter is set to zero because it is redundant;^b^ Dependent variable: CFU.

**Table 6:** *S. aureus* ATCC 6538 and *S. enterica* ATCC 14028 behavior estimates of incubation time (hour) fixed effects with 95% confidence intervals^b^.

Parameter	*Staphylococcus aureus* ATCC 6538							*Salmonella enterica* subsp*. enterica* serovar typhimurium ATCC 14028						
	Estimate	SE	df	*t*	Sig.	LB	UB	Estimate	SE	df	*t*	Sig.	LB	UB
Intercept	3.632	0.289	90	12.565	0.000^*∗∗∗*^	3.058	4.206	3133.002	452.338	90	6.926	0.000^*∗∗∗*^	2234.353	4031.651
Time	−0.144	0.021	90	−6.68	0.000^*∗∗∗*^	−0.187	−0.101	1077.153	639.702	90	1.684	0.000^*∗∗∗*^	−193.727	2348.035
Trial 1	1.455	0.408	90	3.56	0.807	0.643	2.267	1263.938	639.702	90	1.976	0.639	−6.942	2534.819
Trial 2	1.121	0.408	90	2.743	0.306	0.309	1.933	1091.692	639.702	90	1.707	0.632	−179.188	2362.573
Trial 3	1.084	0.408	90	2.652	0.430	0.272	1.896	818.011	639.702	90	1.279	0.595	−452.869	2088.892
Trial 4	0.8132	0.408	90	1.989	—	0.000	1.625	0^a^	0	—	—	—	—	—
Trial 5	0^a^	0	—	—		—	—	−110.793	33.894	90	−3.269	0.002^*∗∗*^	−178.131	−43.456
Trial 1 × time	0.456	0.03	90	14.885	0.049	0.395	0.516	277.036	47.934	90	5.780	0.006	181.806	372.266
Trial 2 × time	−0.007	0.03	90	−0.245	0.009^*∗*^	−0.068	0.053	−22.531	47.934	90	−0.470	0.050	−117.76	72.698
Trial 3 × time	−0.031	0.03	90	−1.03	0.007^*∗∗*^	−0.092	0.029	−23.058	47.934	90	−0.481	0.041	−118.288	72.17
Trial 4 × time	−0.0242	0.03	90	−0.793	0.004^*∗*^	−0.085	0.036	−25.565	47.934	90	−0.533	0.031	−120.795	69.664
Trial 5 × time	0^a^	0	—	—	0.001	—	—	0^a^	0	—	—	0.010	—	—

SE : Standard error; df: degrees of freedom; *t*: the student *t*-statistic; Sig.: the *p* value (associated with the correlation); LB : Lower bound. UB : Upper bound; Trial 1: negative control; Trial 2 : 1 × MIC. Trial 3 : 2 × MIC; Trial 4 : 4 × MIC and Trial 5 : 8 × MIC; ^*∗*^*p* < 0.05; ^*∗*^*p* < 0.01; ^*∗∗∗*^*p* < 0.001, ^a^This parameter is set to zero because it is redundant; ^b^Dependent variable: CFU.

**Table 7:** Estimates of covariance parameters in *S. aureus* ATCC 6538 and *S. enterica* ATCC 14028 behavior estimates of incubation time (A) and trial (B) fixed effect^a^.

		*Staphylococcus aureus* ATCC 6538						*Salmonella enterica* subsp. *enterica* serovar typhimurium ATCC 14028					
		Estimate	SE	Wald Z	Sig.	LB	UB	Estimate	SE	Wald Z	Sig.	LB	UB
A trial fixed	Residual	5.379	0.982239	5.477	0.000	3.761580	7.694	1.788	0.326	5.477	0.000	1.25	2.557
	Time [subject = id] variance	0.000^a^	0.000	0.000	0.015	—	—	0.000^a^	0.000	0.000	0.018	—	—

B time fixed	Residual	0.278	0.082	3.354	0.001	0.155110	0.499	0.278	0.082955	3.354	0.001	0.155	0.499
	Trial [subject = id] variance	0.278^a^	0.000	0.815	0.038	—	—	0.2780^a^	0.000000	0.000	0.029	—	—

^a^Dependent variable: CFU.

## Data Availability

All data used to support the findings of this study are included within the article.
